# Real-world survival of colon cancer after radical surgery: A single-institutional retrospective analysis

**DOI:** 10.3389/fonc.2022.914076

**Published:** 2022-09-16

**Authors:** Xiangyi Pang, Benjie Xu, Jie Lian, Ren Wang, Xin Wang, Jiayue Shao, Shuli Tang, Haibo Lu

**Affiliations:** Department of Outpatient Chemotherapy, Harbin Medical University Cancer Hospital, Harbin, China

**Keywords:** colon cancer, radical surgery, survival rate, nomogram, prognostic factors

## Abstract

The survival rate for colon cancer after radical surgery has been the focus of extensive debate. To assess the postoperative survival and prognostic factors for overall survival (OS), we collected clinicopathological information for 2,655 patients. The survival time and potential risk factors for OS were analyzed by using Kaplan–Meier curves, Cox proportional hazards models, best subset regression (BSR), and least absolute shrinkage and selection operator (LASSO). The 5-year survival rates of stage I–IV colon cancer were 96.6%, 88.7%, 69.9%, and 34.3%, respectively. Adjuvant chemotherapy improved the survival rate (90.4% vs. 82.4%, with versus without adjuvant chemotherapy, respectively) in stage II patients with high-risk factors. Elevated preoperative carcinoembryonic antigen (CEA) and carbohydrate antigen 19-9 (CA19-9) were significantly associated with worse OS compared with patients without these elevations. Less than 12 versus more than 12 harvested lymph nodes (LNs) affected prognosis (84.6% vs. 89.7%, respectively). Regarding the lymph node ratio (LNR), the 5-year OS rate was 89.2%, 71.5%, 55.8%, and 34.5% in patients with LNR values of 0, 0.3, 0.3–0.7, and >0.7, respectively. We constructed a nomogram comprising the independent factors associated with survival to better predict prognosis. On the basis of these findings, we propose that stage II colon cancer patients without high-risk factors and with both elevated preoperative CEA and CA199 should receive adjuvant therapy. Furthermore, the LNR could complement TNM staging in patients with <12 harvested LNs. Our nomogram might be useful as a new prognosis prediction system for colon cancer patients.

## Introduction

Colon cancer is one of the most prevalent malignancies and the second leading cause of cancer-related death in the world (1, 2). According to the latest statistics, there are approximately 520,000 new cases and 250,000 deaths in 2018 in China (3). Currently, the main treatment for colon cancer is radical surgical resection and adjuvant chemotherapy. The 5-year overall survival (OS) rate after radical surgery for colon cancer is approximately 60% in China (4), and the mortality rate remains high. Various factors might be associated with 5-year survival, such as stage, differentiation, histology, tumor location, age, and sex.

Accurate pathological diagnosis, radical surgery, and adequate postoperative adjuvant chemotherapy are critical for colon cancer patients. There have been extensive studies evaluating how to predict the postoperative OS of colon cancer; however, the results are controversial. Therefore, we retrospectively reviewed the clinicopathological data of 2,655 colon cancer patients after radical surgery between January 2011 and December 2016 at Harbin Medical University Cancer Hospital. A nomogram was developed based on clinical and histopathological high-risk factors to determine the OS of colon cancer patients undergoing curative resection.

## Materials and methods

The clinical data of 2,655 colon cancer patients treated between January 2011 and December 2016 were derived from the Department of Colorectal Surgery of Harbin Medical University Cancer Hospital. Patients who received palliative surgery, had multiple primary cancers, or received neoadjuvant chemotherapy were excluded; patients lost to follow-up were also excluded (**Supplementary Figure 1**). All patients were randomly divided into a training cohort and a testing cohort at a ratio of 70% to 30%. This study was approved by the Harbin Medical University Cancer Hospital ethics committee (KY2017-19), and written informed consent was obtained from the patients.

We evaluated the following baseline covariates: sex, age, pathological tumor (pT) stage, pathological lymph node (pN) stage, pathological metastasis (pM) stage, tumor location, tumor size, histology, differentiation, examined lymph nodes (LNs), metastatic lymph node ratio (LNR), perineural invasion, lymphatic invasion, preoperative carcinoembryonic antigen (CEA) level, preoperative carbohydrate antigen 199 (CA199) concentration, and adjuvant chemotherapy. Cancers of the proximal splenic flexure (cecum and ascending colon) were classified as right colon cancer. Cancers in the distal splenic flexure (descending colon and sigmoid colon) were classified as left colon cancer. Cancer in the middle of the transverse colon was classified as transverse cancer. Regarding adjuvant therapy, patients were classified into two groups (i.e., with vs. without). Patients were also categorized into three groups by their age at the time of the primary tumor resection, as follows: <40 years, 40–70 years, and >70 years. CEA level was classified as ≤5 ng/ml or >5 ng/ml based on the upper normal limit (5 u/ml), and CA199 level was classified as ≤37 U/ml or >37 U/ml based on the upper normal limit (37 U/ml). Tumor size was categorized as <5 cm or ≥5 cm. All patients in this study were reclassified in accordance with the 8th TNM classification of colon cancer.

All statistical analyses were performed using SPSS software (version 25.0; IBM Corp., Armonk, NY, USA). OS analyses were performed using the Kaplan–Meier (KM) method, and the results were compared using the log-rank test. Clinical and pathological variables associated with survival were assessed on the basis of clinical importance, scientific evidence, and predictors identified in previously published articles (5, 6). The variables screened by univariate Cox regression, best subset regression (BSR), and least absolute shrinkage and selection operator (LASSO) were included in a multivariate Cox regression model, and the final model of the three methods was determined using backward stepwise selection with minimum Akaike’s information criterion (AIC) values (**Supplementary Table 1**). Candidate variables with *p*-values < 0.2 in the univariate analysis were included in the multivariable model. Risk factors were measured as [hazard ratio (HR), 95% confidence interval (CI)]. All reported statistical significance levels were two-sided, with statistical significance set at 0.05. The predictive nomogram was developed based on a multivariate analysis using the rms package in R, version 4.1.2 (www.r-project.org). Calibration plots, Harrell’s concordance index (C-index), area under the curve (AUC) values, and decision curve analysis (DCA) were used to evaluate calibrating ability.

## Results

### Clinicopathological characteristics

**Figure 1:**
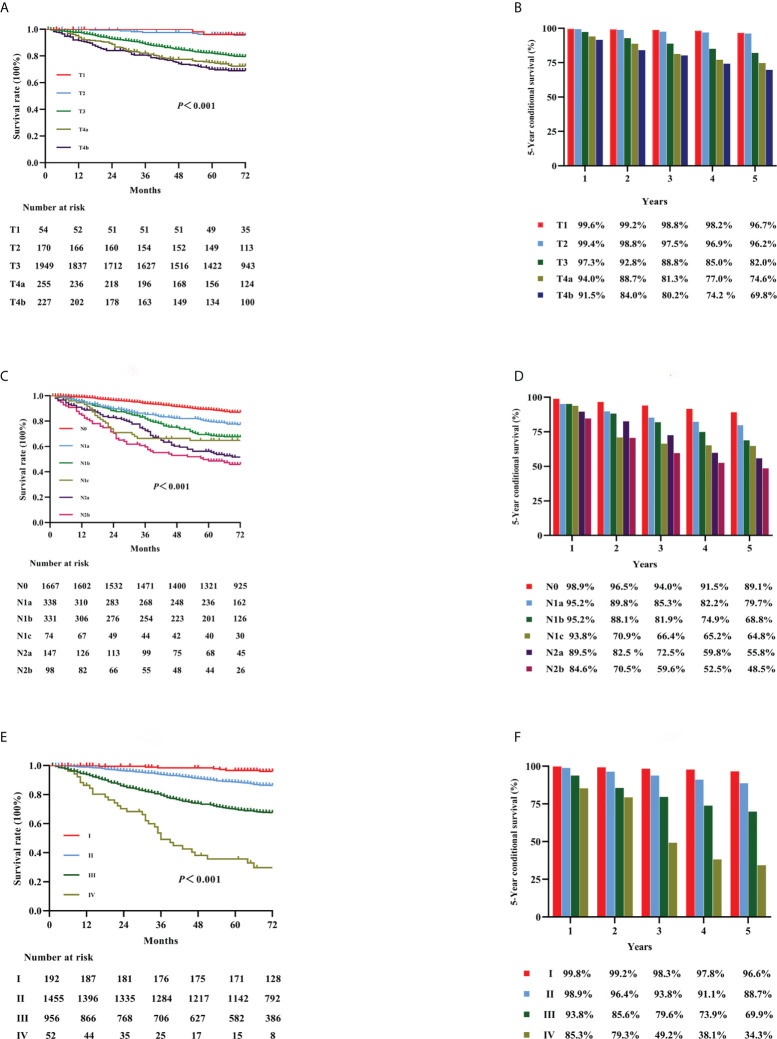
Kaplan–Meier curves for survival analysis in colon cancer patients. The OS according to T stage **(A)**, N stage **(C)**, and the TNM staging system **(E)**. The 5-year OS rates of the three classifications [T stage, N stage, and TNM staging: **(B, D, F)**, respectively]. OS, overall survival; T, tumor; N, node; TNM, tumor-node-metastasis.

The baseline clinicopathological characteristics of the patients are shown in **Table 1**. More than half of the patients had tumors larger than 5.0 cm (*N* = 1,740, 65.5%). Regarding the differentiation grade of the primary site, approximately 9.0%, 81.5%, 5.2%, and 4.3% of the colon cancer patients had well-differentiated, moderately differentiated, poorly differentiated, and undifferentiated tumors, respectively. The proportions of stage T1, T2, T3, and T4 cancer were 2.0%, 6.2%, 73.4%, and 18.2%, respectively. Stages II and III were more prevalent (*N* = 2,411, 90.8%) than stages I and IV, and 62.8% of the patients were classified as N0 (**Figure 1**). Regarding the LNs, approximately 70% of the patients had sufficient LNs examined (≥12; *N* = 1995, 75.1%), and the average number of LNs examined was 18. At the end of the follow-up, the mortality rate was 20.6%, and the median survival was 67.94 months.

**Table 1:** Clinical and pathological characteristics of the study population.

Characteristics	*N* (%)	Five-year OS rate
Sex		
Male	1,511 (56.9)	82.2
Female	1,144 (43.1)	80.7
Age at diagnosis		
<40	123 (4.6)	87.6
40–70	2,060 (77.6)	83.5
>70	472 (17.8)	71.3
Preop CEA (ng/L)		
≤5	1,620 (61.0)	85.8
>5	971 (36.6)	74.6
Missing	64 (2.4)	
Preop CA199 (ng/L)		
≤37	2,165 (81.5)	84.2
>37	398 (15.0)	68.0
Missing	92 (3.5)	
Tumor location		
Right	1,186 (44.7)	80.4
Transverse	66 (2.5)	84.0
Left	1,403 (52.8)	82.4
Tumor size (cm)		
<5	915 (34.5)	85.5
≥5	1,740 (65.5)	79.4
Histology		
Adenocarcinoma	1,814 (68.3)	83.0
Mucinous adenocarcinoma	57 (2.1)	67.0
Mixed cell adenocarcinoma	784 (29.5)	79.1
Differentiation		
Well differentiated	239 (9.0)	90.1
Moderately differentiated	2,163 (81.5)	81.9
Poorly differentiated	139 (5.2)	71.5
Undifferentiated	114 (4.3)	69.2
8th T stage		
T1	54 (2.0)	96.1
T2	170 (6.4)	95.6
T3	1,949 (73.4)	82.0
T4	482 (18.2)	72.7
8th N stage		
N0	1,667 (62.8)	89.1
N1	743 (29.0)	73.5
N2	245 (9.2)	53.1
8th M stage		
M0	2,603 (92.7)	82.4
M1	52 (7.3)	35.7
Examined nodes		
<12	1,995 (75.1)	82.2
≥12	660 (24.9)	79.4
LNR		
LNR = 0	1,667 (62.8)	89.1
0 < LNR < 0.3	792 (29.8)	72.7
0.3 ≤ LNR < 0.7	163 (6.1)	55.0
LNR ≥ 0.7	33 (1.2)	34.5
Perineural invasion		
Negative	2,110 (79.5)	84.4
Positive	359 (13.5)	63.7
Missing	186 (7.0)	
Vascular invasion		
Negative	2,247 (84.6)	82.8
Positive	222 (8.4)	67.2
Missing	186 (7.0)	
Adjuvant chemotherapy		
Yes	1,110 (41.8)	83.5
No	1,545 (58.2)	80.1

OS, overall survival; CA199, carbohydrate antigen 19-9; CEA, carcinoembryonic antigen; LNR, lymph node ratio; T, tumor; N, node; M, metastasis.

### Univariate and multivariate analyses

**Figure 2:**
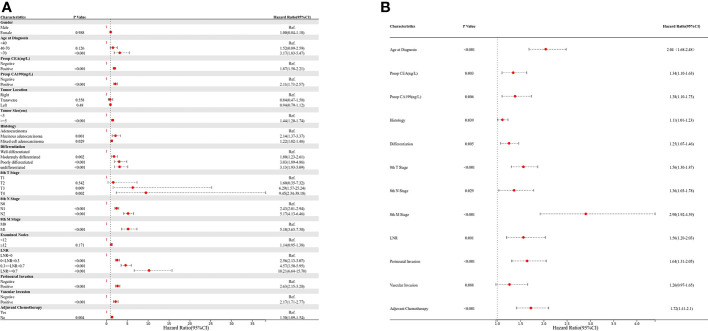
Univariate and multivariate Cox regression analyses; **(A)** univariate analysis; **(B)** multivariate analysis.

Univariate and multivariate regression analyses were performed to determine the prognostic factors in colon cancer patients after curative surgical resection (**Figures 2A, B**). Age, preoperative CEA and CA199 levels, tumor size, histology, differentiation, pT stage, pN stage, M stage, number of harvested LNs, LNR, perineural invasion, vascular invasion, and adjuvant chemotherapy were identified as significant factors correlated with OS in the univariate analysis (**Figure 2A**). The multivariate analysis showed that age, preoperative CEA and CA199 levels, histology, differentiation, pT stage, pN stage, M stage, LNR, perineural invasion, and adjuvant chemotherapy were independent prognostic factors (**Figure 2B**).

### Five-year OS and TNM stage

KM curves for stages T1, T2, T3, T4a, and T4b in all patients are shown in **Figure 1A** (*p* < 0.001). The OS rates for stages T1, T2, T3, T4a, and T4b were 96.7%, 96.2%, 82.0%, 74.6%, and 69.8%, respectively (**Figure 1B**). KM curves for stages N0, N1a, N1b, N1c, N2a, and N2b for all patients are shown in **Figure 1C** (*p* < 0.001). The OS rates for stages N0, N1a, N1b, N1c, N2a, and N2b were 89.1%, 79.7%, 68.8%, 64.8%, 55.8%, and 48.5%, respectively (**Figure 1D**). KM curves in accordance with the 8th edition TNM staging for stage I, II, III, and IV cancer are shown in **Figure 1E** (*p* < 0.001). The OS rates for cancer stages I, II, III, and IV were 96.6%, 88.7%, 69.9%, and 34.3%, respectively (**Figure 1F**).

### Preoperative CEA and CA199

**Figure 3:**
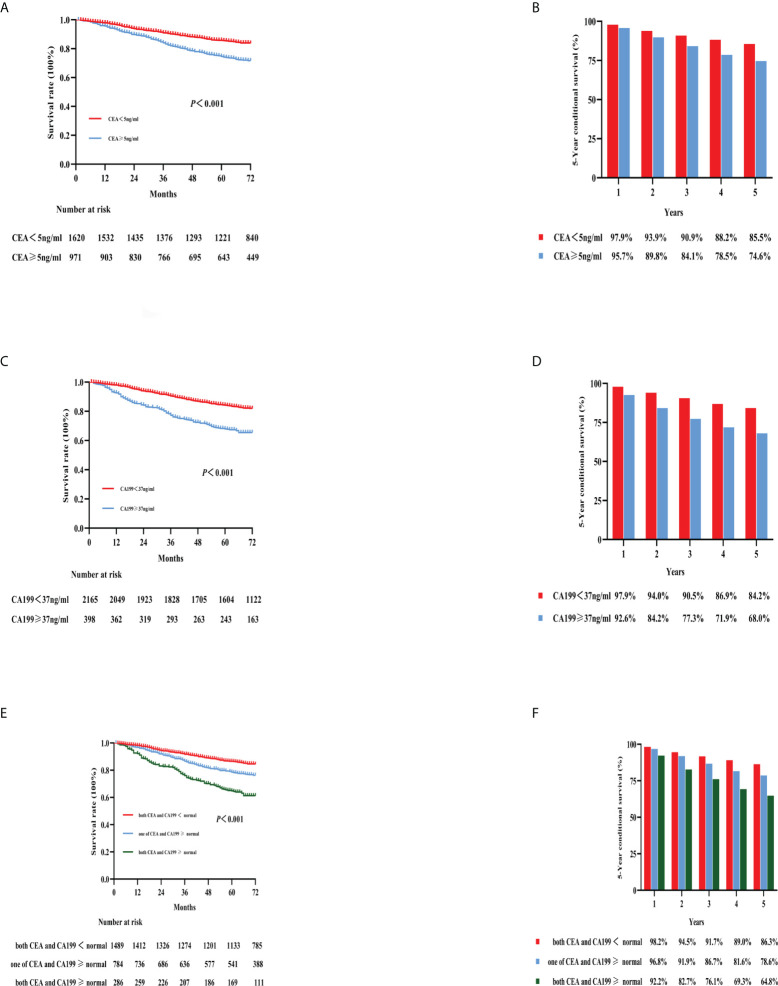
Kaplan–Meier curves for the survival analysis according to the level of preoperative CEA **(A)**, CA199 **(C)**, and combined CEA and CA199 **(E)**. The 5-year OS rates of the three categories are shown in **(B, D, F)**: CEA, CA199, and combined CEA and CA199, respectively. CEA, carcinoembryonic antigen; CA199, carbohydrate antigen 199; OS, overall survival.

For all patients, elevated preoperative CEA (5-year OS: 85.5% vs. 74.6%; *p* < 0.001) level or CA19-9 (5-year OS: 84.2% vs. 68.0%; *p* < 0.001) level was significantly associated with worse OS versus no elevation in either of these markers, respectively (**Figures 3A–D**). Furthermore, patients with both elevated CEA and CA199 (*N* = 286, 10.8%) had the worst 5-year OS compared with patients with other groups (5-year OS: 64.8%; *p* < 0.001) (**Figures 3E, F**).

### Risk factors in stage II colon cancer patients who received chemotherapy

**Figure 4:**
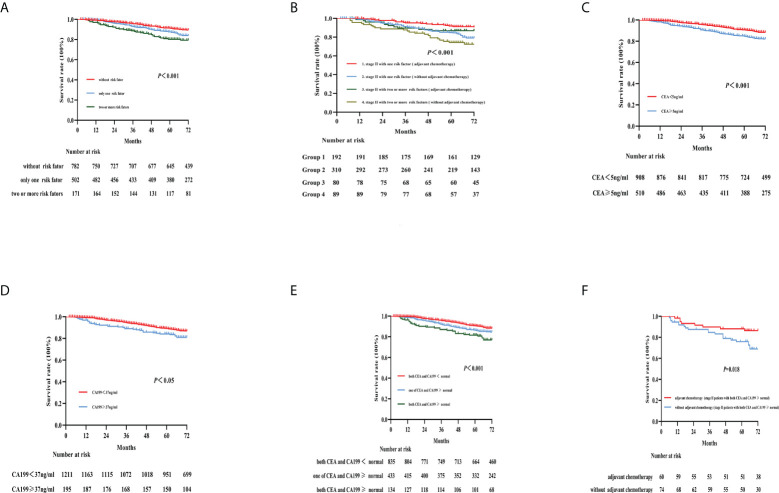
Kaplan–Meier curves for the survival analysis in stage II colon cancer patients. The figures show OS according to different risk factors **(A)**, adjuvant chemotherapy with different risk factors **(B)**, preoperative CEA and CA199 levels **(C–E)**, and adjuvant chemotherapy combined with elevated CEA and CA199 **(F)**. OS, overall survival; CEA, carcinoembryonic antigen; CA199, carbohydrate antigen 199.

In this study, stage II patients accounted for more than half of the cases (*N* = 1,455, 54.8%). The baseline clinicopathological characteristics of the patients with stage II colon cancer are shown in **Supplementary Table S2**. These patients were divided into three groups based on the number of risk factors (7, 8), which were stage pT4, bowel obstruction or tumor perforation, high tumor grade, vascular or perineural invasion, and <12 LNs examined. Accordingly, patients were classified as without risk factors (*N* = 782), only one risk factor (*N* = 502), or two or more risk factors (*N* = 171), and the 5-year OS rates were 91.2%, 87.3%, and 80.2%, respectively (**Figure 4A**). The 5-year survival of patients with deficient mismatch repair (dMMR) colon cancer was better than that in patients with proficient MMR (pMMR) without high-risk factors (5-year OS: 96.5% vs. 88.6%, respectively; *p* = 0.040) (**Supplementary Figure 2A**). The same trend was not observed in patients with high-risk factors (5-year OS: 88.0% vs. 87.6%, dMMR vs. dMMR, respectively; *p* = 0.687) (**Supplementary Figure 2B**). Additionally, in this study, we analyzed the effect of adjuvant chemotherapy on the 5-year OS rate of stage II colon cancer in different risk factor groups. In the group with only one risk factor, adjuvant chemotherapy improved OS (5-year OS: 91.5% vs. 84.6%, with versus without adjuvant chemotherapy, respectively; *p* < 0.001). Similar findings were observed for patients with two or more risk factors (5-year OS: 84.2% vs. 73.5%, with versus without adjuvant chemotherapy, respectively; *p* < 0.001) (**Figure 4B**). Preoperative CEA and CA199 levels had effects on survival and prognosis in patients with stage II colon cancer (**Figures 4C, D**). Elevated preoperative CEA and CA199 levels were associated with the worst 5-year OS rates (81.5% for both; *p* < 0.001) (**Figure 4E**). Adjuvant chemotherapy significantly improved the survival rate of patients without high-risk factors and with both elevated preoperative CEA and CA199 (*p* = 0.018) (**Figure 4F**).

### Harvested LNs in stage II/III colon cancer and LNR

**Figure 5:**
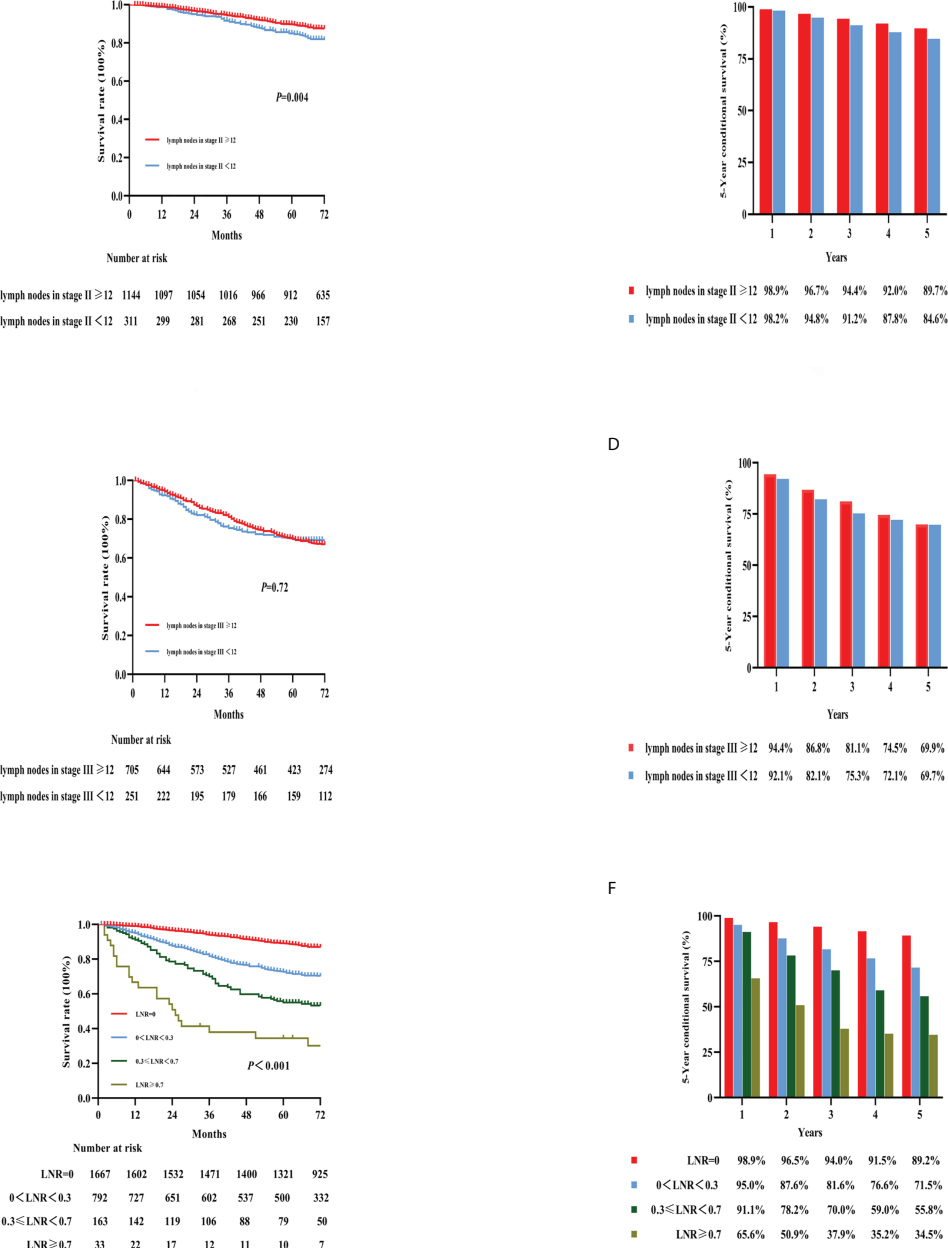
Kaplan–Meier curves for the survival analysis according to the number of examined lymph nodes. The figures show OS in stage II colon cancer patients **(A)**, stage III colon cancer patients **(C)**, LNRs **(E)**, and the 5-year OS rates of the three categories [**(B, D, F)**, respectively]. OS, overall survival; LNR, lymph node ratio.

The National Comprehensive Cancer Network (NCCN) guidelines recommend that at least 12 LNs be examined after surgery (9). Our study analyzed how the number of harvested LNs affected 5-year OS. The 5-year OS rate was associated with a significantly different prognosis in patients with stage II colon cancer with different numbers of examined LNs (**Figures 5A, B**; 5-year OS: LNs > 12: 89.7%, LNs < 12: 84.6%; *p* < 0.001). However, there was no significant difference for patients with stage III colon cancer regarding the number of examined LNs (**Figures 5C, D**; 5-year OS: 69.9% vs. 69.7%, >12 LNs vs. <12 LNs, respectively; *p* = 0.72).

LNR was defined as the proportion of the number of positive LNs to the number of harvested LNs. In our previous study (10), 0.3 and 0.7 were determined as the best cutoff values for LNR. Therefore, we divided the patients in this study into four groups with cutoff values of 0, 0.3, 0.3–0.7, and 0.7; the respective 5-year OS rates were 89.2%, 71.5%, 55.8%, and 34.5% (**Figures 5E, F**). Furthermore, no clear survival difference was observed between adequate examined LNs (≥12 LNs) and inadequate examined LNs (<12 LNs) in the same LNR group (**Supplementary Figure 3**).

### Construction of the nomogram

**Figure 6:**
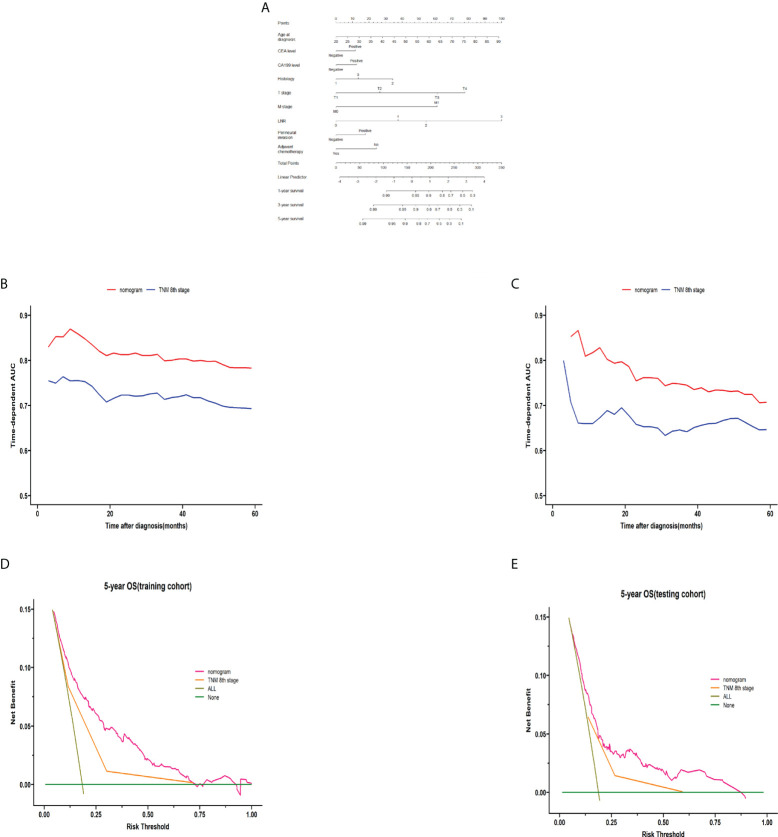
Construction and validation of the nomogram. The nomogram for predicting 1-, 3-, and 5-year survival in colon cancer **(A)**. A web-based tool based on the formulated nomogram is available at https://coloncancernomogram20220118.shinyapps.io/DynNomapp/. The time-dependent AUCs using the nomogram to predict 5-year overall survival probability in both the training and testing cohorts are shown in panels **(B, C)**, respectively. Decision curve analysis of the nomogram and TNM 8th edition staging for the survival prediction of patients with colon cancer are also shown. The 5-year survival benefit in both the training and testing cohorts is shown in panels **(D, E)**, respectively. For LNR, 0 indicates LNR = 0, 1 indicates LNR = 0–0.3, 2 indicates LNR = 0.3–0.7, and 2 indicates LNR >0.7. For the histology, 1 indicates adenocarcinoma, 2 indicates mucinous adenocarcinoma, and 3 indicates mixed cell adenocarcinoma. AUC, area under the curve; TNM, tumor-node-metastasis; LNR, lymph node ratio.

On the basis of the stepwise regression results, the model containing age, CEA, CA199, histology, pT stage, pM stage, LNR, perineural invasion, and adjuvant chemotherapy had the minimal AIC value in the training cohort. A nomogram for colon cancer was constructed based on the results of the final multivariable model. **Figure 6A** shows an example of using the nomogram to predict the survival probability of a given patient. In the training cohort, the AUC was 0.783 at 5 years (**Supplementary Figure 4A**), and the C-index was 0.759. The OS nomogram was validated internally, which indicated an AUC at 5 years of 0.713 (**Supplementary Figure 4B**) and a C-index of 0.703. **Supplementary Figure 5** shows the bootstrapped calibration plots in the training and testing cohorts. The discrimination ability indicated that in the training as well as testing sets, the nomogram was better than the TNM 8th staging classification for predicting survival. In both the training and testing cohorts (**Figures 6B, C**), the time-dependent AUC was >0.7 for the prediction of OS within 5 years. The time-dependent C-index was >0.7 for the prediction of OS within 5 years in both the training and validation cohorts (**Supplementary Figure 6**). DCA curves showed that the nomogram was better at predicting 5-year OS compared with the TNM 8th staging classification (**Figures 6D, E**).

## Discussion

In our study, the OS rates for stage I, II, III, and IV colon cancer were 96.6%, 88.7%, 69.9%, and 34.3%, respectively. Stage I and II survival rates were significantly higher than those for stage III and IV, which was consistent with previous studies (11). This difference might be related to tumor invasion and cell infiltration (12–14). To improve the survival of colon cancer patients, it is necessary to focus on prevention and early detection and treatment.

The key prognostic factors for colon cancer, especially the high-risk factor of stage II disease, have been a hot topic of research. The poor prognosis for stage II colon cancer has been confirmed to be associated with high-risk factors (15, 16). In our study, the presence of a higher number of high-risk factors was confirmed to be associated with worse OS based on the risk factor grouping. Additionally, adjuvant chemotherapy improved the survival rate in stage II patients with high-risk factors in our study. This is consistent with the results of earlier studies, including the QUASAR trial (17) and some retrospective analyses (18, 19). The recommendation that patients with stage II colon cancer with high-risk factors should receive postoperative adjuvant chemotherapy is also consistent with the current guidelines (20). Previous studies have shown that stage II patients with dMMR have a better prognosis than stage II patients with pMMR. However, another study indicated that pMMR should not be the only risk factor considered when determining whether to administer adjuvant chemotherapy in patients with stage II colon cancer or not (21).

The prognostic role of CEA and CA199 in predicting survival in colon cancer patients has been frequently studied; however, there is currently no reliable evidence confirming this role (22). Our study found that preoperative CEA and CA199 levels were independent prognostic factors in colon cancer. Elevated preoperative CEA and CA19-9 levels were significantly associated with worse OS. Furthermore, patients with both elevated CEA and CA199 levels had the worst 5-year OS rate. The same results were seen in stage II colon cancer patients. In a recent study, researchers found that CEA and CA199 were independent predictors of colon cancer recurrence and survival (23). However, there is no randomized controlled trial to evaluate whether adjuvant chemotherapy should be recommended for stage II patients without high-risk factors and with both elevated preoperative CEA and CA199. Our findings suggested that high preoperative levels of CEA and CA199 significantly affected the prognosis and survival of stage II colon cancer patients and that these factors can be high-risk factors for the need for chemotherapy in stage II colon cancer. This finding agrees with the results of a retrospective analysis by Thirunavukarasu et al. (24). There are also other clinical or pathological factors that significantly affect prognosis and survival in stage II patients.

Another issue that we evaluated in this study was the number of LNs examined. Pathological N staging reflects a strong association between the number of positive LNs and survival (6, 25). Notably, in this study, the 5-year OS rate for stage II patients with an insufficient number of harvested LNs (< 12) was 84.6%, while the 5-year OS rate for patients with a sufficient number of harvested LNs was 89.7%. However, there was no similar trend regarding LNs in stage III patients. This means that an insufficient number of LNs were examined, resulting in undetected positive LNs, especially in patients with stage II colon cancer. To avoid missing positive LNs, the NCCN guidelines recommend that the number of LNs collected should be more than 12 (9), which was further validated in our study.

A growing number of studies have shown that LNR is associated with prognosis, and that this ratio can be used as an independent prognostic tool to assess the prognosis of colon cancer patients (26, 27). However, currently, the optimal cutoff value for LNR is controversial. In our previous study, an information gain method was developed to redefine the cutoff value for LNR. The thresholds were 0, 0–0.3, 0.3–0.7, and >0.7. The study was based on a large sample of the Surveillance, Epidemiology and End Results database (SEER) to redefine the cutoff value for LNR (10). The thresholds of LNR were proved to be better than the previously reported studies (28, 29). Based on thresholds of 0, 0.3, and 0.7, the respective 5-year OS rates were 89.2%, 71.5%, 55.8%, and 34.5% in our study. There were different pN stages in each LNR group. In the LNR (0.3–0.7) group, we found that patients with pN1 stage colon cancer had worse 5-year OS than patients with pN2 stage cancer. Further observations showed that less than 12 LNs examined led to an underestimation of the pN stage. Moreover, the predictive effect of LNR was independent of the number of LNs examined, and other studies have found similar results (30, 31).

In this study, we developed and validated a nomogram for the postoperative individualized prediction of survival in colon cancer patients. The nomogram incorporates age, CEA, CA199, histology, pT stage, pM stage, LNR, perineural invasion, and adjuvant chemotherapy. Integrating the clinical and pathological risk factors into a convenient nomogram helps predict postoperative survival. It is important to perform an accurate risk stratification for patients with cancers such as colon cancer because the prognosis may be heterogeneous (32, 33). Nomograms may afford a more individualized method to provide prognostic information for patients compared with the TNM staging system. Compared with the TNM staging system, our nomogram had better performance for risk stratification of the prognosis of patients undergoing radical colon cancer surgery, with better AUC values and C-index.

## Conclusions

TNM staging is indispensable for postoperative survival assessment of colon cancer and for determining whether postoperative adjuvant therapy is needed. Our results showed that elevated preoperative CEA and CA199 levels are poor prognostic factors in patients with colon cancer. Preoperative CEA and CA199 elevations should be considered as risk factors in stage II colon cancer patients. We propose that stage II colon cancer patients without high-risk factors and with both ele-vated preoperative CEA and CA199 should receive adjuvant therapy. Future randomized controlled trials to evaluate the role of adjuvant chemotherapy in these patients are needed. To avoid missing positive LNs and subsequent inadequate staging of LNs metastases, it is recommended that at least 12 lymph nodes be examined. LNR can be used as a supplement to pN staging when the number of LNs examined is insufficient. The nomogram described in this study predicted the survival of colon cancer patients better than that when using the TNM system.

Our study was a retrospective study performed in a single center. Although the study involved a large data sample, a study of colon cancer diagnosis, surgery, and postoperative treatment from a single center has limitations that may lead to selection bias. Hopefully, more data from multiple centers and larger samples will lead to more convincing conclusions.

## Data availability statement

The raw data supporting the conclusions of this article will be made available by the authors, without undue reservation.

## Ethics statement

The studies involving human participants were reviewed and approved by Harbin Medical University Cancer Hospital ethics committee (KY2017-19). The patients/participants provided their written informed consent to participate in this study.

## Author contributions

HL and JL contributed to the study conception and design. XP, BX, RW, and XW collected the data. XP and BX analyzed the data. XP, BX, JS, and ST interpreted the data. XP and BX drafted the manuscript. HL and JL contributed to critical revision of the manuscript. All authors read and approved the final manuscript.

## Funding

This work was supported by the National Natural Science Foundation of China (grant nos. U20A20376 and 61972116) Beijing Award Foundation (grant nos. YXJL-2020-0818-0478) Wu Jieping Medical Foundation (grant no. 320.6750.2020-19-20) Heilongjiang Province Postdoctoral Science Foundation(grant no. LBHZ21189) Harbin Medical University Innovative Science Research Funded Project (grant no. 31041220028) China Postdoctoral Science Foundation(grant no. 2022MD713747).

## Acknowledgments

We thank Jane Charbonneau, DVM, from Liwen Bianji (Edanz) (www.liwenbianji.cn) for editing the English text of a draft of this manuscript.

## Conflict of interest

The authors declare that the research was conducted in the absence of any commercial or financial relationships that could be construed as a potential conflict of interest.

## Publisher’s note

All claims expressed in this article are solely those of the authors and do not necessarily represent those of their affiliated organizations, or those of the publisher, the editors and the reviewers. Any product that may be evaluated in this article, or claim that may be made by its manufacturer, is not guaranteed or endorsed by the publisher.
