# Supplementary material for: Real-world survival of colon cancer after radical surgery: A single-institutional retrospective analysis
Source: Front Oncol. 2022 Sep 16;12:914076. doi: 10.3389/fonc.2022.914076 (PMC9525022; doi:10.3389/fonc.2022.914076)
Supplement: Supplementary file 1 [file DataSheet_1.docx]

Supplementary Material

# Supplementary Figures and Tables

## Supplementary Figures


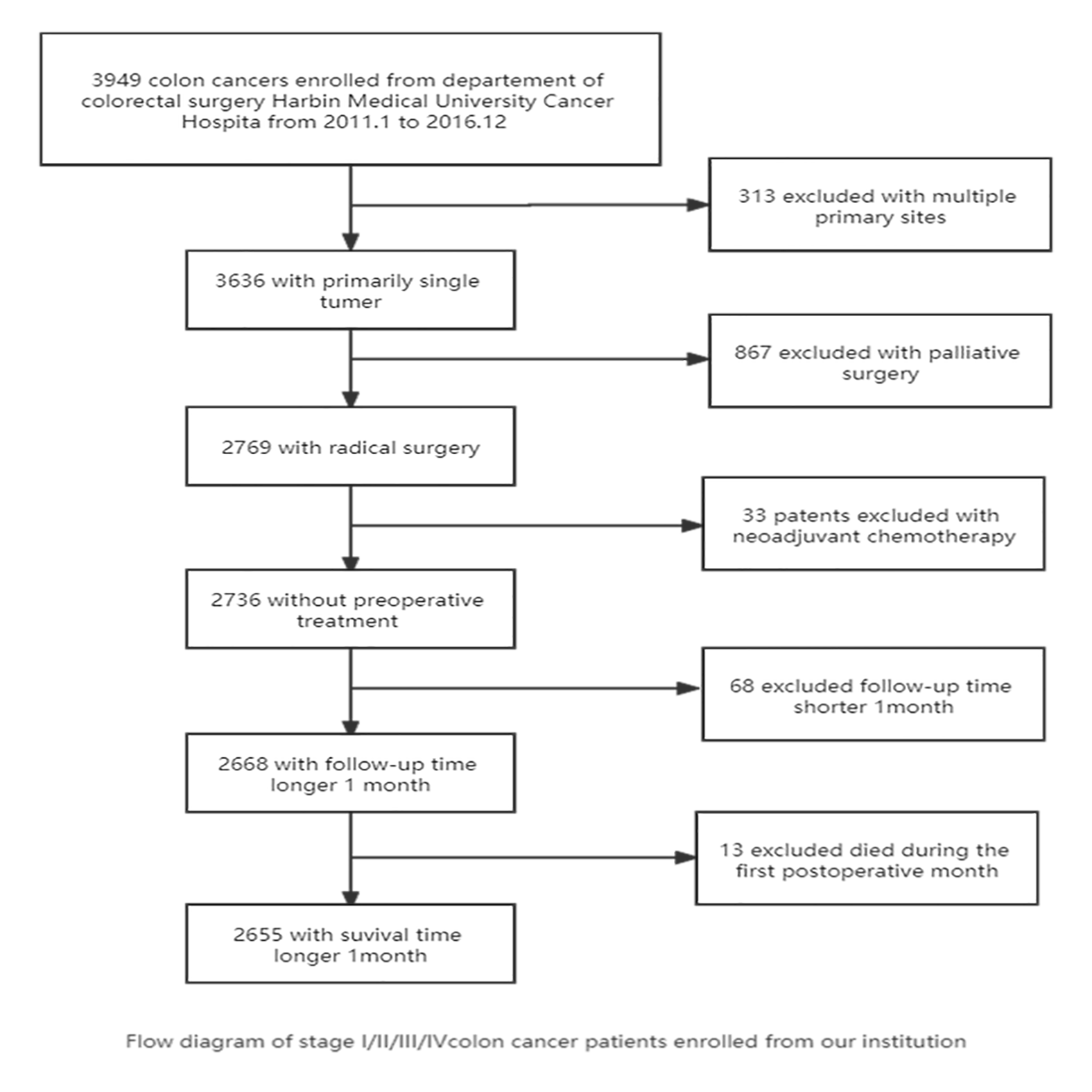


**Supplementary Figure 1.** Flow diagram of all patients after curative resection for colon cancer enrolled from Harbin Medical University Cancer Hospital between January 2011 and December 2016.


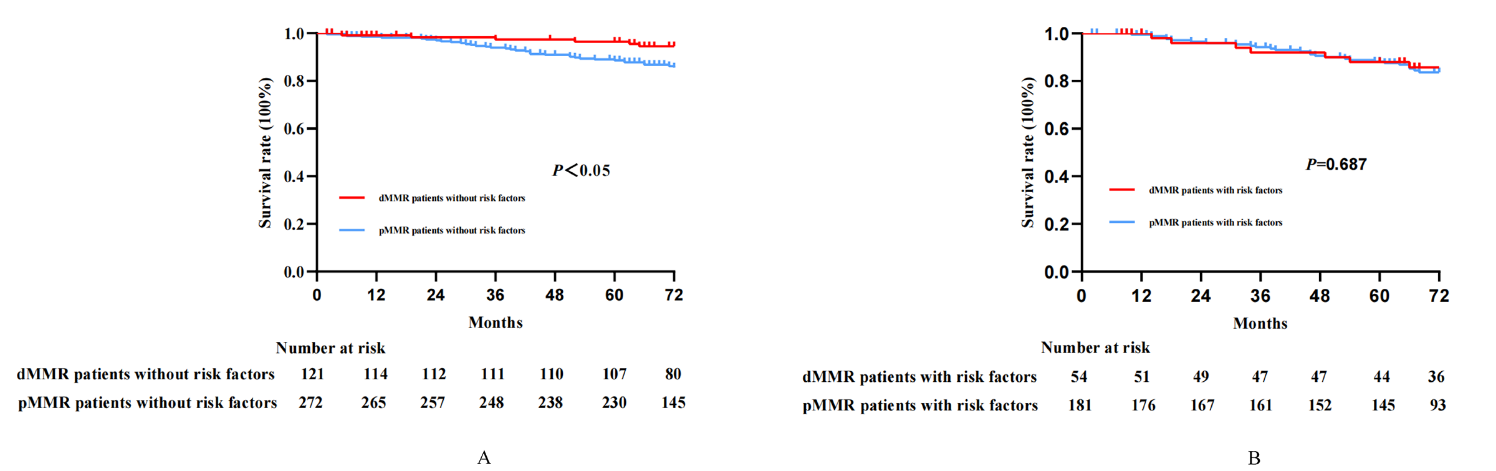


**Supplementary Figure 2.** Kaplan–Meier curves of the two sets. The figures show OS in patients without risk factors **(A)**, patients with risk factors **(B)**.

dMMR, deficient mismatch repair; pMMR, proficient mismatch repair.


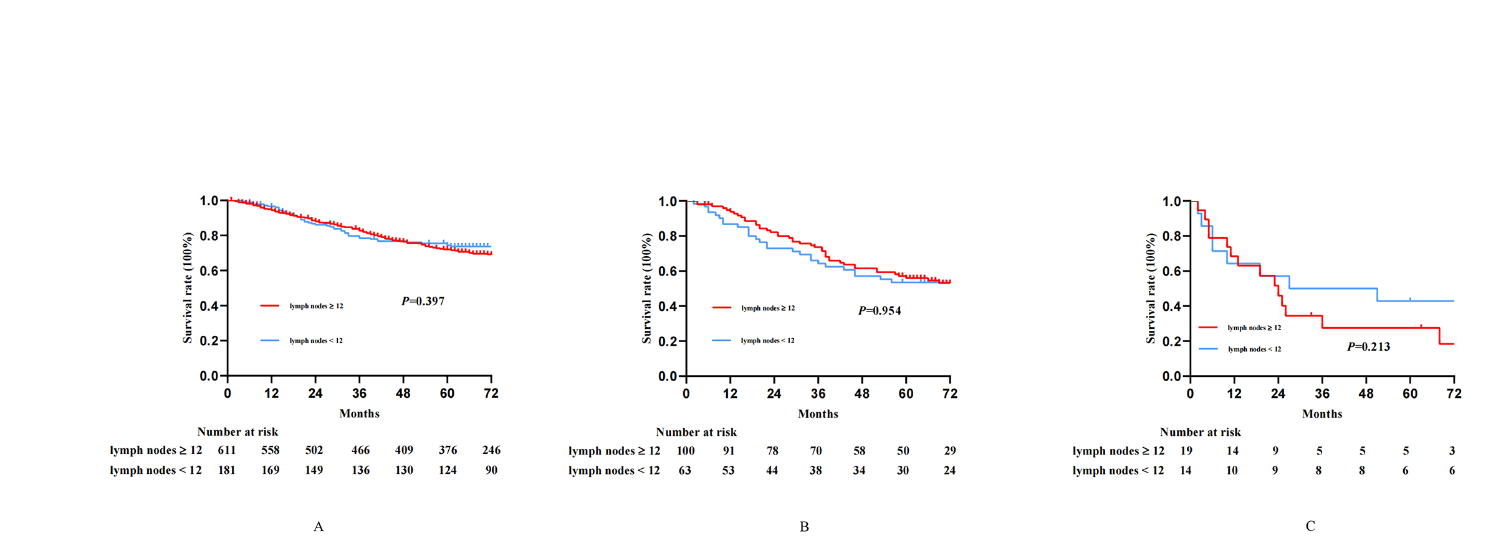


**Supplementary Figure 3.** Kaplan–Meier curves of the two sets. The figures show OS in LNR = 0–0.3 **(A)**, LNR = 0.3–0.7 **(B)**, LNR >0.7 **(C).**

OS, overall survival.


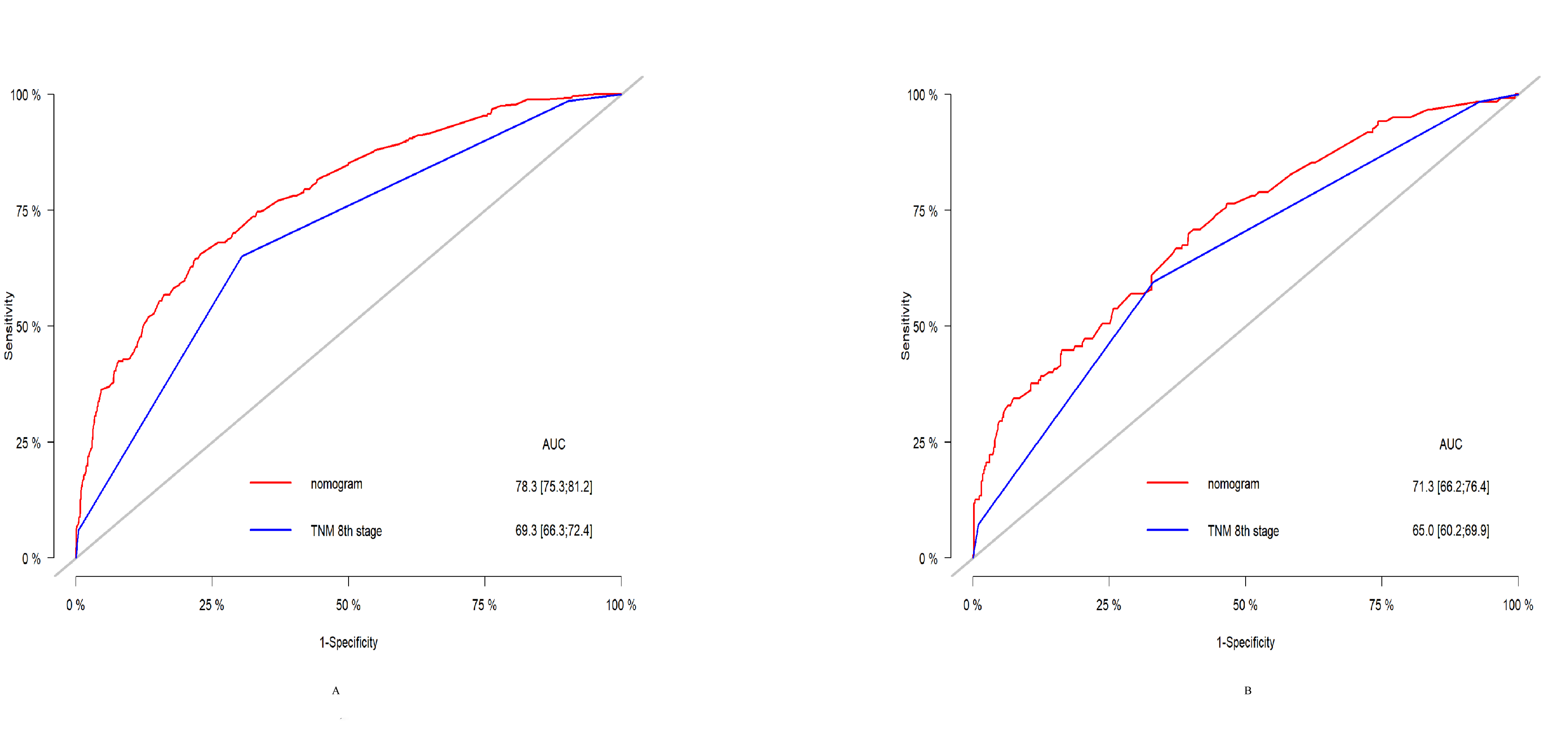


**Supplementary Figure 4.** The ROC curves of the nomogram and TNM 8th edition staging with 5-year OS in both the training and testing cohorts **(A, B)**.

ROC, receiver operating characteristic curve; OS, overall survival.


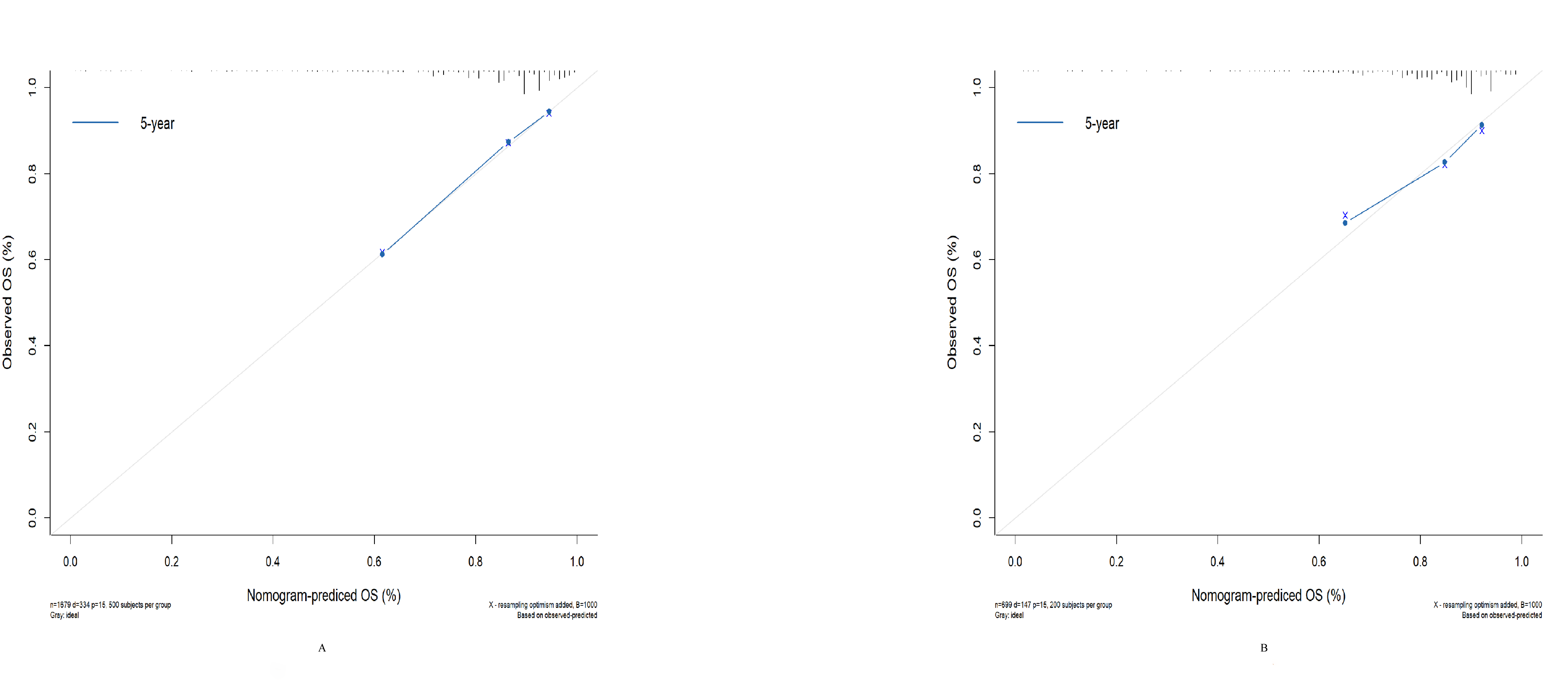


**Supplementary Figure 5.** Calibration plots of 5-year OS determined using the nomograms for both the training and testing cohorts **(A, B)**.

OS, overall survival.


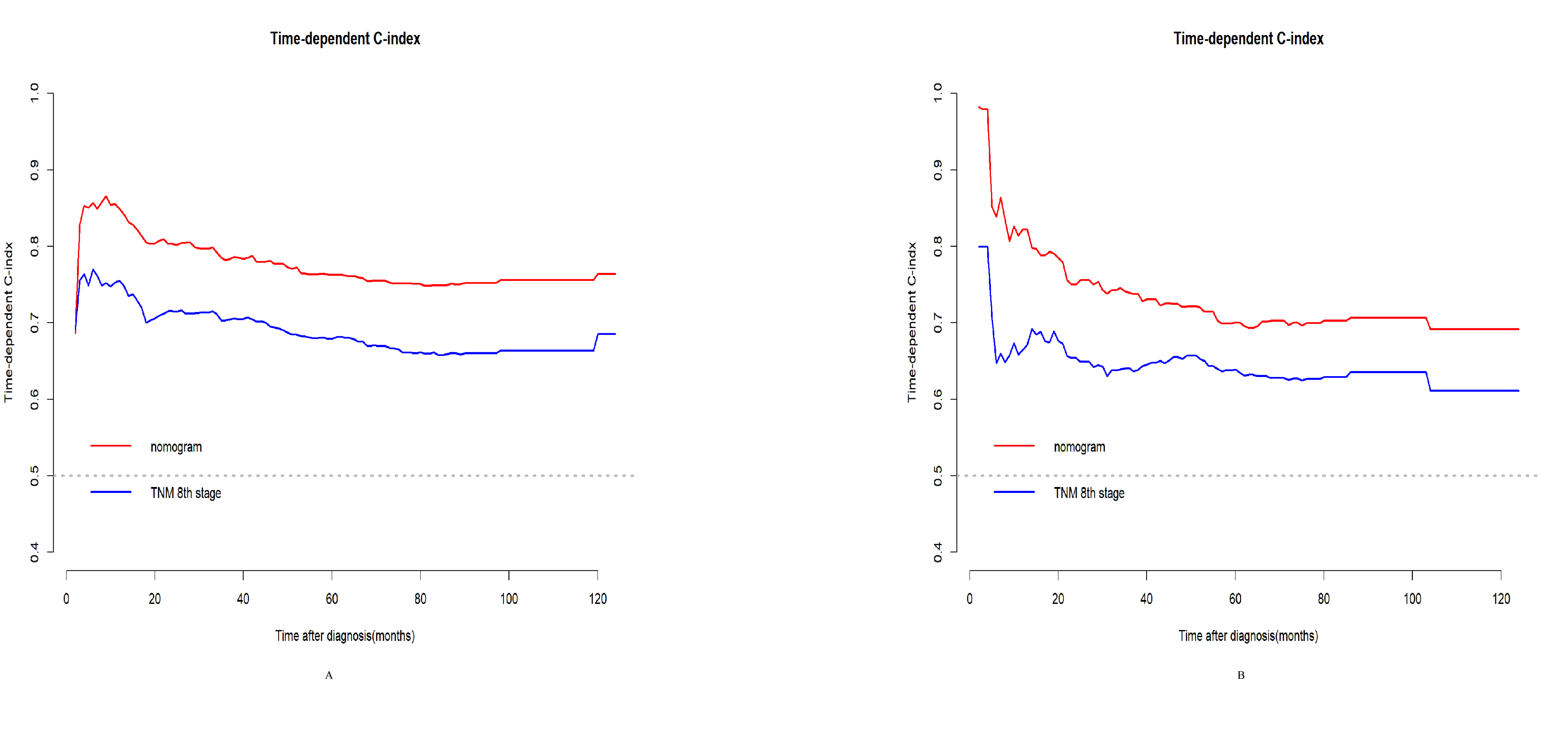


**Supplementary Figure 6.** Time-dependent C-index using the nomogram to predict 10-year OS probability in both the training and testing cohorts (**(A)** and **(B)**).

OS, overall survival.

## Supplementary Tables

| **Method** | **AIC** |
| --- | --- |
|  |  |
| Univariate Cox Regression | 4494.44 |
| BSR | 4533.3 |
| LASSO | 4529.35 |
| AIC, Akaike information; BSR, best subset regression; LASSO least absolute shrinkage and selection operator.  **Supplementary Table 1.** The minimum AIC value for the variables screened using three methods in the multivariate Cox regression analysis.  AIC, Akaike information   \| **Characteristics** \| **n (%)** \| \| --- \| --- \| \| \| **Sex** \|  \| \| Male \| 851(58.5) \| \| Female \| 604(41.5) \| \| **Age at Diagnosis** \|  \| \| <40 \| 73(5.0) \| \| 40-70 \| 1105(75.9) \| \| ＞70 \| 277(19.0) \| \| **Preop CEA** (ng/L) \|  \| \| ≤5 \| 908(62.4) \| \| ＞5 \| 510(35.1) \| \| Missing \| 37(2.5) \| \| **Preop CA199**(ng/L) \|  \| \| ≤37 \| 1211(83.2) \| \| ＞37 \| 195(13.4) \| \| Missing \| 49(3.4) \| \| **Tumor Location** \|  \| \| Right \| 716(49.2) \| \| Transverse \| 44(3.0) \| \| Left \| 695(47.8) \| \| **Tumor Size**(cm) \|  \| \| <5 \| 429(29.5) \| \| ≥5 \| 1026(70.5) \| \| **Histology** \|  \| \| Adenocarcinoma \| 957(65.8) \| \| Mucinous adenocarcinoma \| 34(2.3) \| \| Mixed cell adenocarcinoma \| 464(31.9) \| \| **Differentiation** \|  \| \| Well differentiated \| 137(9.4) \| \| Moderately differentiated \| 1191(81.9) \| \| Poorly differentiated \| 63(4.3) \| \| undifferentiated \| 64(4.4) \| \| **8^th^ T Stage** \|  \| \| T3 \| 1195(82.1) \| \| T4 \| 260(17.9) \| \| **Examined Nodes** \|  \| \| <12 \| 311(21.4) \| \| ≥12 \| 1144(78.6) \| \| **Perineural Invasion** \|  \| \| Negative \| 1218(83.7) \| \| Positive \| 135(9.3) \| \| Missing \| 102(7.0) \| \| **Vascular Invasion** \|  \| \| Negative \| 1288(88.5) \| \| Positive \| 65(4.5) \| \| Missing \| 102(7.0) \| \| **MMR Status** \|  \| \| pMMR \| 453(31.1) \| \| dMMR \| 175(12.0) \| \| Missing \| 827(56.8) \| \| [**Adjuvant Chemotherapy**](https://dict.bioon.com/m/detail.asp?id=00e92076993) \|  \| \| Yes \| 516(35.5) \| \| No \| 939(64.5) \| \| Abbreviations: CA199, carbohydrate antigen 19-9; CEA, carcinoembryonic antigen; dMMR, deficient mismatch repair; pMMR, proficient mismatch repair. \| \|   **Supplementary Table 2.** Clinical and pathological characteristics of stage II colon cancer patients. | |
